# Validation of Polish Version of Dispositional Flow Scale-2 and Flow State Scale-2 Questionnaires

**DOI:** 10.3389/fpsyg.2022.818036

**Published:** 2022-04-25

**Authors:** Justyna Józefowicz, Natalia Kowalczyk-Grębska, Aneta Brzezicka

**Affiliations:** ^1^The Faculty of Information Technology, Polish-Japanese Academy of Information Technology, Warsaw, Poland; ^2^The Faculty of Psychology, SWPS University of Social Sciences and Humanities, Warsaw, Poland

**Keywords:** flow, positive psychology, scale adaptation, multidimensional instruments, validation, flow dimensions

## Abstract

The aim of this study is to evaluate the psychometric properties of the Polish version of the Dispositional Flow Scale-2 (DFS-2) and Flow State Scale-2 (FSS-2), for use with Polish adults and young adults. Currently, there are no tools that would allow us to study flow among Polish speakers. At the same time, due to the great interest in flow and its potential importance for effectiveness, cooperation, and learning, it is worth ensuring that reliable validated measurement questionnaires are available for people studying the Polish population. Study participants completed 856 questionnaires, of which 496 individuals (with an average age of 36.31 years) participated in the DFS-2 study and 360 individuals (with an average age of 33.46 years) participated in the FSS-2 study. The maximum likelihood estimator (MLR) was selected for the CFA analysis. Model fit was assessed using: χ2, comparative fit index (CFI), Tucker-Lewis index (TLI), and standardized root mean square of residuals (SRMR), and root mean square of approximation error (RMSEA). For both questionnaires, formative first-level models with nine factors and second-level models with nine factors loaded on a higher-order flow factor were compared using the Satorra-Bentler Scaled difference χ2 test. The ω coefficient was used to estimate the reliability of the FFS-2 and DFS-2 models tested in the CFA method. Confirmatory factor analysis of both DFS-2 structural models showed satisfactory model fit. Most of the fit indices for the hierarchical 2nd order FSS-2 model presented satisfactory values, except for SRMR. Both DFS-2 and FSS-2 factors tested in the analysis showed good reliability (ω ≥ 0.7). Our findings confirmed the reliability and validity of the Polish versions of DFS-2 and FSS-2 scales. The scales are reliable when applied to Polish adults and young adults.

## Introduction

### Essence of Flow

Flow is a construct defining how the subject describes the subjective experience of engaging in challenging activity. It represents a basic psychological state that allows one to experience a full life. It can occur in any area of life (Csíkszentmihályi, 1990; Moneta and Csíkszentmihályi, 1996) and is related to the satisfaction derived from performing various activities. Dealing with the challenge was made real through a series of goals, receiving continuous feedback on progress and modifying actions based on this information (Nakamura and Csíkszentmihályi, 2009).

From the perspective of positive psychology, an understanding of the concept of flow can be considered crucial, because being completely absorbed in what one is doing is a desirable state of human functioning and the essence of the “good life” (Nakamura et al., 2002), and experiencing flow leads to personal growth (Seligman and Csíkszentmihályi, 2000).

### Dimensions of Flow

Csíkszentmihályi (1990) and Moneta and Csíkszentmihályi (1996) distinguished and described nine dimensions (three conditions and six characteristics), each of which represents a distinctive conceptual dimension of the flow experience, while together they represent the flow experience (Jackson and Eklund, 2002). Csikszentmihalyi considered the balance between the challenge of the task and the skills of the individual to be the primary “universal precondition for flow” (Csíkszentmihályi and Csíkszentmihályi, 1992), thus broadening the perspective, as it was originally thought that optimal experience depends mainly on this condition (Engeser and Rheinberg, 2008; Landhäußer and Keller, 2012). For the balance to be maintained, clarity of goals and direct feedback are essential. So there are three conditions:

1. *Challenge-Skill Balance* (a sense of the adequacy of one’s competence and ability and the level of challenge in what one is doing).
2. *Clear Goals* (feeling confident about the action one is taking),
3. *Unambiguous Feedback* (immediate and clear feedback).

If these conditions are met it becomes possible to:

1. *Merging of Action and Awareness* (deep involvement makes one act spontaneously and almost automatically),
2. *Concentration on the Task at Hand* (a sense of complete focus),
3. *Sense of Control* (feeling that you can handle the situation),
4. *Loss of Self-Consciousness* (lack of a sense of concern for oneself and one’s performance),
5. *Transformation of Time* (feeling that the way time passes is distorted),
6. *Autotelic Experience* (experience of an activity in which the mere fact of doing that activity is satisfying).

### Flow Research. A Historical Perspective

Research on the flow as a subjective state characterized by the intensity of the level of attention experienced by people while performing intrinsically motivated activities began in the mid-seventies (Csíkszentmihályi, 1975). So far, flow has been studied mainly in sports, music, chess, dance (Csíkszentmihályi and Larson, 1987; Csíkszentmihályi and Csíkszentmihályi, 1992; Csíkszentmihályi, 1993, 2000, 2014), or recently also in the context of video games (Nah et al., 2014).

Flow experience is both relevant and ephemeral and it is a key construct of positive psychology (Nakamura et al., 2002). Currently, researchers are continuing to search and construct testing of measurement tools most relevant to flow measurement. These include: The Flow Questionnaire (Nakamura and Csíkszentmihályi, 2009; Moneta, 2012), Experience Sampling Method (ESM) (Nakamura and Csíkszentmihályi, 2009; Moneta, 2012; Csíkszentmihályi and Larson, 2014), Flow State Scale-2 (FSS-2), Dispositional Flow Scale-2 (DFS-2) (Jackson and Eklund, 2002; Moneta, 2012), Flow Short Scale (Delle Fave et al., 2011b; Engeser, 2012), Flow Scale Mayersa (Nakamura and Csíkszentmihályi, 2009), Optimal Experience Survey (Cuestionario de Experiencia Óptima; CEO) (Delle Fave et al., 2011a).

### Dispositional Flow Scale-2, Flow State Scale-2

The most frequently used and approved are the Dispositional Flow Scale-2 (DFS-2) and the Flow State Scale (FSS-2) (Kawabata et al., 2008).

For their study of sport and physical activity, Jackson and Marsh (1996) transformed Csikszentmihalyi’s nine-factor flow model into the Flow State Scale (FSS). Composed of 36 questions, the FSS was designed to assess the experience of flow during a specific activity, with data collected immediately after the activity (Jackson and Marsh, 1996).

Flow as a trait and tendency to experience flow was measured by the Dispositional Flow Scale (DFS), which was an extension of the Flow Trait Scale (TFS), also developed for measurement among athletes (Jackson et al., 1998). The DFS is a dispositional measure of flow and is used to assess the typical frequency of experiencing flow during participation in a particular activity.

The results of preliminary analyzes of the psychometric properties of DFS and FSS showed that both tools showed a satisfactory level of factor validity (Jackson and Marsh, 1996; Marsh and Jackson, 1999) and reliability (e.g., 1–4 0.72–0.91 for FSS and 1/4 0.70–0.88 for DFS, Jackson et al., 1998). Middleton et al. (2004) also reported an acceptable reliability (1/4 0.71–0.86) of DFS.

Reacting to the fact that some items in the original tool did not give satisfactory results (from a conceptual and/or statistical point of view), Jackson and Eklund (2002) revised both tools, replacing the problematic items and developed new versions of the flow scales: Flow State Scale-2 (FSS-2) and Dispositional Flow Scale-2 (DFS-2). Nine-dimensional conceptualization of flow, and the nine-factor structure was supported by confirmatory factor analyses (Jackson and Eklund, 2002). The higher order model, representing the global flow construct, also received reasonable support from confirmatory factor analysis (CFA).

For the nine subscales of the DFS and FSS adequate reliability has been demonstrated, with the exception of time and self-awareness, which showed lower internal consistency (Jackson and Marsh, 1996; Marsh and Jackson, 1999; Tenenbaum et al., 1999; Kawabata et al., 2008; Gouveia et al., 2012).

Finally, Jackson et al. (2008b) published FSS-2 and DFS-2 in two versions: a. a long version with 36 items, divided equally into nine factors, corresponding to the nine dimensions of Csíkszentmihályi (1990) and b. a short version consisting of nine items, one for each dimension.

FSS-2 and DFS-2 have been translated and validated in various languages, especially often in the context of sports (Doganis et al., 2000, Fournier et al., 2007; Calvo et al., 2008; Kawabata et al., 2008; Gonzalez-Cutre et al., 2009; Gouveia et al., 2012; Crust and Swann, 2013; Riva et al., 2017; Nojavan et al., 2017), but also learning, recreation, well-being (Whitmore and Borrie, 2005; Asakawa, 2010, Rufi et al., 2014, Souza Costa Correia et al., 2020), gameplay and internet games (Wang et al., 2009; Procci et al., 2012; Hamari and Koivisto, 2014), mental diseases (Huang et al., 2019).

Most previous studies have used exploratory factor analysis rather than CFA. Kawabata et al. (2008) point out the difficulty of interpreting the expression of subjective experience without cross-cultural and cross-linguistic biases. The present study aimed to develop psychometrically valid Polish versions of the FSS-2 and DFS-2 (PFSS-2 and PDFS-2).

Currently, there are no tools to measure the flow in Polish culture. The purpose of this study is to remedy this deficiency. Providing Polish researchers with access to reliable tools for flow measurement seems to be particularly important due to the possibility of filling gaps in the analysis of processes related to the functioning of individuals and teams in various contexts, with particular emphasis on the processes of learning and development. A promising indication here are research on flow as a mediator of the relationships between attentional control and approaches to studying (Cermakova et al., 2010), as a mediator between psychological ownership and employees’ subjective happiness (Fan et al., 2019), or considering the essence of flow as a mediator or moderator in the context of the impact of flow on relationship between resources and organizational outcomes (Seifert, 2015).

### Flow in Video Games

One of the areas in which flow has been studied is games and gamification, i.e., the application of game mechanics to non-game areas (Wang et al., 2009; Procci et al., 2012; Hamari and Koivisto, 2014). Games, due to the complexity of activities, are a kind of flow base. The player is in a positive mental state, so focused on the game that nothing else matters. The activity is not necessarily aimed at a specific benefit. It is a sense of total presence, of detachment from reality–without fear or hope. It is an autotelic action, that is, it provides value to the player in itself. In complex activities that require a lot of cognitive, motor, or temporal input, flow seems to be a particularly important and quite natural state. On the other hand, the level of involvement of the person and the autotelicity that this phenomenon generates lead researchers to look at flow in the context of processes that are desirable for learning, building motivation and engagement in the activities undertaken. The interest in flow in games can be evidenced by the numerous studies on the topic that have emerged in the last year alone (Gao and Lu, 2021; Gutierrez, 2021; Cai et al., 2022; Jogo et al., 2022, etc.).

In addition to its general purpose of providing Polish researchers with a reliable tool for studying such an important phenomenon as flow, the present study was prompted by the specific need to allow for the examination of participants’ flow while learning a complex video game in a large study focused on the effects of video games on cognitive function.

## Materials and Methods

### The Validation Process

After verifying the presence of tools to measure the state and predisposition of flow in the Polish language, we found that at the moment there are no tools measuring flow in the Polish culture. As part of the initial consultation, we looked at several questionnaires (Nakamura and Csíkszentmihályi, 2009; Delle Fave et al., 2011; Engeser, 2012; Moneta, 2012; Csíkszentmihályi and Larson, 2014). The most frequently used and approved are the Dispositional Dispositional Flow Scale-2 (DFS-2) and the Flow State Scale (FSS-2), a reliable and validated tool with a long tradition in many countries, for instance: France (Fournier et al., 2007), Spain (Rufi et al., 2014), Italy (Riva et al., 2017), China (Huang et al., 2019), Greece (Stavrou and Zervas, 2004).

Flow–both as a disposition and a state–can affect both the learning process and the work of individuals (Cermakova et al., 2010; Seifert, 2015; Fan et al., 2019) and teams (Hout et al., 2017). Providing Polish researchers with access to tools for studying flow (as a moderator and mediator) is a step that is not only needed but also a necessity.

The translation process was carried out in three stages: 1. Establishment of a committee of experts, 2. translation into Polish, 3. backwards translation. The FSS-2 and DFS-2 questionnaires in the English version were translated by two translators, verified, and then translated backwards by a native speaker and re verified by psychologists.

The next step was to assess the psychometric properties of the questionnaire, in which 496 people (DFS-2) and 360 people (FSS-2) were used in such Polish language versions.

For the purposes of the study, a license was obtained to use both questionnaires (contact the authors of the questionnaires, Mind Garden, Inc. 18 March 2019) and the guidelines for their use “The Flow Scales Instrument and Scoring Guide” by Jackson et al. (2008a), published by Mind Garden, Inc.

### Materials

Dispositional Flow Scale 2 (DFS-2) and Flow State Scale 2 (FSS-2) were developed by Jackson and Eklund (2002, 2004), based on previous tools developed by Jackson and Marsh (1996). These 36-point scales were developed to assess the experience of flow at disposition and state level based on the Csíkszentmihályi (1990) nine-dimensional flow concept.

Both scales are a set of 36 items, consisting of 4 items for each of the 9 flow dimensions: *Challenge skill balance, Merging of Action and Awareness, Clear goals, Unambiguous feedback, Concentration on the Task at Hand, Sense of control, Loss of self-consciousness, Transformation of time, Autotelic experience.*

Participants were asked to indicate the degree of their agreement with each of the elements as characterizing their disposition (DFS-2) and their experience with the activity just ended (FSS-2) on a “Likert-type” scale ranging from 1 (strongly disagreeing) to 5 (strongly agreeing).

### Participants

Two independent groups of participants took part in the study: a. participants who agreed to participate in the DFS-2 study, b. participants who agreed to participate in the FSS-2 study.

Some participants agreed to both measurements.

Both questionnaires were completed by Polish-speaking people from all over Poland (big city, town, village) over 18 years old. Due to the limitations of COVID-19, the survey was conducted in an online format, so participants had to have access to the Internet. For FSS-2, they were ready to participate in a 1.5-h workshop where they played the online game *Symbols*. Participants, having previously read the study agenda, signed an informed consent, and then proceeded to play the game. For the DFS-2, the task was simpler, requiring only the completion of a questionnaire. Participants made their own decisions about which of the two studies they chose to engage in.

Recruitment for the study was conducted through online channels, student, volunteer, business, educational, and development networks. In addition to those included in the study, 17 individuals (12K, 5M) who did not meet the basic eligibility requirements were not included in the study: individuals under 18 years of age (11K, 2M), non-Polish speakers (1K, 3M). Participants were recruited for research using social media and information on websites.

### Demographic Data

The research was attended by 856 persons (Table 1), of which 496 participants (female *N* = 286, male *N* = 210) were qualified for the DFS-2 tool, and 360 persons (female *N* = 190, male *N* = 169, other *N* = 1) for the FSS-2 questionnaire survey tool.

**TABLE 1:** Demographic data of the participants.

	DFS-2 (*n* = 496)	FSS-2 (*n* = 360)
*M* (*SD*) Age in years	36.31 (10.97)	33.46 (10.03)
**Sex**		
Male	210 (42.3%)	169 (24.6%)
Female	286 (57.6%)	190 (52.8%)
Other	0 (0%)	1 (0.03%)
**Place of residence**		
Village	134 (27.0%)	75 (20.8%)
Town	137 (27.6%)	201 (55.8%)
City	225 (45.4%)	84 (23.4%)

The participants of the survey are residents of:

for DFS-2: villages (*N* = 134), towns (*N* = 137), and cities (*N* = 225),
for FSS-2: villages (*N* = 75), towns (*N* = 201), and cities (*N* = 84).

Due to the large disproportion between the number of applications from people with higher education and primary education (98,2%–higher education), it was decided to limit the group to people with higher education or in the process of learning only (secondary education).

### Course of the Study

Participants belonging to the first group (a.), having been previously informed about the study, signed the informed consent and filled in the Polish version of the DFS-2 questionnaire.

Participants belonging to the second group (b.), having previously read the study agenda, signed the informed consent and then proceeded to play the game “Symbols”, in groups of 15–30 people, online. After completing the task, participants completed the FSS-2 questionnaire, indicating flow as a state.

Participants played “Symbols” online. Each participant received (virtually) two cards with symbols. Only the owner of the cards could see their cards. The group’s goal was to arrange the set of cards in the only possible position on the game board. The first step was to figure out, using only verbal communication, how to do it (45 min), the second step was to guide the instructor to put the cards in the right place according to the participants’ instructions (3 min). Each participant was responsible for telling the instructor where to place each card.

The game “Symbols” was chosen among several games that were considered because of the intensity and fluctuating involvement of the participants.

Standard informed consent procedures were followed during data collection and institutional approval for the study was obtained (Ethical Committee Opinion No: 6/2021 issued by the Research Ethics Committee of the Faculty of Psychology, SWPS University in Warsaw).

### Statistics

#### Confirmatory Factor Analyses

The robust maximum likelihood estimator (MLR) was chosen for the CFA as it was shown to produce adequate estimation of factor loadings for 5-point Likert scales (Rhemtulla et al., 2012). Model fit was evaluated based on the following indices: χ2, comparative fit index (CFI), Tucker-Lewis index (TLI), and standardized root mean square residual (SRMR), and root mean square error of approximation (RMSEA). The following thresholds were considered metrics of a satisfactory model fit: CFI > 0.9, TLI > 0.9, SRMR < 0.8, and RMSEA < 0.08 (Hair et al., 2010). Preferably, the χ2 test should not be significant. Nevertheless, it is not necessary since the χ2 test is based on sample size (Russell, 2002).

For both questionnaires, we compared the formative first level models with nine factors and second level models with nine factor loaded by a higher order flow factor (Jackson and Eklund, 2002) with the use of a Satorra-Bentler Scaled difference χ2 test (Satorra and Bentler, 2010). All analyses were calculated using R software (R Core Team, 2021), with the use of lavaan (Rosseel, 2012), and semTools (Jorgensen et al., 2021) packages.

#### Reliability Analyses

Coefficient ω was used to estimate the reliability of FFS-2 and DFS-2 models tested in CFA ((Dunn et al., 2014; Flora, 2020).

## Results

### Confirmatory Factor Analysis of the Dispositional Flow Scale-2

Both structural models of DFS-2 presented satisfactory model fit (Table 2), however, the metrics were better for DFS-2 first order model. Moreover, the Satorra-Bentler Scaled difference χ2 showed a significant difference between the models, χ2 difference = 162.91, *p* < 0.001, favoring the formative first level model with nine factors. The factor loadings of the model are presented in the Table 3.

**TABLE 2:** Fit indices for two tested models of DFS questionnaire.

	χ 2	df	CFI	TLI	RMSEA [90% CI]	SRMR
DFS-2 second order, hierarchical model	1183.52	585	0.931	0.926	0.050 [0.045–0.054]	0.065
DFS-2 first order model	1009.53	558	0.948	0.941	0.044 [0.040–0.048]	0.054

*CFI, comparative fit index; TLI, Tucker-Lewis index; SRMR, standardized root mean square residual; RMSEA, root mean square error of approximation.*

**TABLE 3:** Factor loadings and reliability measures for the Dispositional Flow Scale-2 models tested in the study.

	Factor Loadings and reliability	
	Second order, nine factors	First order, nine factors
**Challenge skill balance (ω = 0.83)**		
1. I am challenged, but I believe my skills will allow me to meet the challenge	0.63	0.63
10. My abilities match the challenge of what I am doing	0.74	0.73
19. I feel I am competent enough to meet the demands of the situation	0.83	0.84
28. The challenge and my skills are at an equally high level	0.75	0.74
**Action and awareness (ω = 0.77)**		
2. I do things correctly without thinking about trying to do so	0.59	0.53
11. Things just seem to happen automatically	0.54	0.53
20. I do things automatically, without thinking too much	0.8	0.74
29. I do things spontaneously and automatically without having to think	0.84	0.85
**Clear goals (ω = 0.88)**		
3. I know clearly what I want to do	0.77	0.74
12. I have a strong sense of what I want to do	0.83	0.84
21. I know what I want to achieve	0.83	0.83
30. My goals are clearly defined	0.77	0.80
**Unambiguous feedback (ω = 0.85)**		
4. It is really clear to me how I am going	0.75	0.72
13. I am aware of how well I am doing	0.83	0.8
22. I have a good idea about how well I am doing while I am involved in the task/activity	0.88	0.82
31. I can tell by the way things are progressing how well I am doing	0.73	0.71
**Task concentration (ω = 0.80)**		
5. My attention is focused entirely on what I am doing	0.82	0.75
14. It is no effort to keep my mind on what is happening	0.19	0.39
23. I have total concentration	0.87	0.82
32. I am completely focused on the task at hand	0.91	0.88
**Sense of control (ω = 0.87)**		
6. I have a sense of control over what I am doing	0.77	0.71
15. I feel like I can control what I am doing	0.83	0.78
24. I have a feeling of total control over what I am doing	0.9	0.83
33. I feel in total control of my actions	0.86	0.8
**Loss of self-consciousness (ω = 0.90)**		
7. I am not concerned with what others may be thinking of me	0.86	0.88
**Transformation of time (ω = 0.83)**		
8. Time seems to alter (either slows down or speeds up)	0.83	0.73
17. The way time passes seems to be different from normal	0.91	0.83
26. It feels like time goes by quickly	0.6	0.63
35. I lose my normal awareness of time	0.82	0.74
**Autotelic experience (ω = 0.88)**		
9. I really enjoy the experience of what I am doing	0.81	0.71
18. I love the feeling of what I am doing and want to capture this feeling again	0.82	0.82
27. The experience leaves me feeling great	0.93	0.85
36. The experience is extremely rewarding	0.66	0.83
**Global flow factor (ω = 0.85)**		
Challenge skill balance	0.89	
Action and awareness	0.56	
Clear goals	0.86	
Unambiguous feedback	0.85	
Task concentration	0.74	
Sense of control	0.92	
Loss of self-consciousness	0.50	
Transformation of time	0.30	
Autotelic experience	0.71	

### Reliability Analysis of the Dispositional Flow Scale-2 Questionnaire

All factors tested in the analysis showed good reliability, as indicated by ω ≥ 0.7 (Table 3).

Additionally, intercorrelations of items of the DFS-2 questionnaire are presented in the Supplementary Material.

### Confirmatory Factor Analysis of the Flow State Scale-2

Most of the fit indices for the second order hierarchical FSS-2 model presented satisfactory values, with an exception of the SRMR (Table 4). The parameters of the model were explored to find what caused the misfit. For the hierarchical model, the factor “*Transformation of time*” was not significantly loaded by the global flow factor (*r* = 0.10, *p* = 0.150). The FSS-2 first order model showed better values of all fit indices. Additionally, the Satorra-Bentler Scaled difference χ2 showed a significant difference between the models, χ2 difference = 92.00, *p* < 0.001, favoring the formative first level model with nine factors. The factor loadings of the model are presented in the Table 5.

**TABLE 4:** Fit indices for the models of the Flow State Scale-2.

	χ 2	df	CFI	TLI	RMSEA [90% CI]	SRMR
FSS-2 second order, hierarchical model	1105.91	585	0.925	0.919	0.056 [0.051–0.061]	0.087
FSS-2 first order model	1007.64	558	0.936	0.927	0.053 [0.048–0.058]	0.075

*CFI, comparative fit index; TLI, Tucker-Lewis index; SRMR, standardized root mean square residual; RMSEA, root mean square error of approximation.*

**TABLE 5:** Factor loadings of the Flow State Scale-2 questionnaire.

	Factor Loadings and reliability	
	Second order, nine factors	First order, nine factors
Challenge skill balance (ω = 0.87)		
1. I was challenged, but I believed my skills would allow me to meet the challenge	0.64	0.64
10. My abilities matched the challenge of what I was doing	0.87	0.86
19. I felt I was competent enough to meet the demands of the situation	0.9	0.9
28. The challenge and my skills were at an equally high level	0.77	0.78
**Action and awareness (ω = 0.80)**		
2. I did things correctly without thinking about trying to do so	0.59	0.59
11. Things just seemed to be happening automatically	0.54	0.55
20. I did things automatically, without thinking too much	0.80	0.8
29. I did things spontaneously and automatically without having to think	0.84	0.83
**Clear goals (ω = 0.88)**		
3. I knew clearly what I wanted to do	0.77	0.76
12. I had a strong sense of what I wanted to do	0.83	0.83
21. I knew what I wanted to achieve	0.83	0.83
30. My goals were clearly defined	0.77	0.78
**Unambiguous feedback (ω = 0.87)**		
4. It was really clear to me how I was going	0.75	0.75
13. I was aware of how well I was doing	0.83	0.83
22. I had a good idea about how well I was doing while I was involved in the task/activity	0.88	0.88
31. I could tell by the way things were progressing how well I was doing	0.73	0.73
**Task concentration (ω = 0.79)**		
5. My attention was focused entirely on what I was doing	0.82	0.82
14. It was no effort to keep my mind on what was happening	0.19	0.18
23. I had total concentration	0.87	0.87
32. I was completely focused on the task at hand	0.91	0.9
**Sense of control (ω = 0.91)**		
6. I had a sense of control over what I was doing	0.77	0.76
15. I felt like I could control what I was doing	0.83	0.84
24. I had a feeling of total control over what I was doing	0.90	0.9
33. I felt in total control of my actions	0.86	0.86
**Loss of self-consciousness (ω = 0.92)**		
7. I was not concerned with what others may have been thinking of me	0.86	0.86
16. I was not concerned with how others may have been evaluating me	0.88	0.88
25. I was not concerned with how I was presenting myself	0.81	0.81
34. I was not worried about what others may have been thinking of me	0.91	0.91
**Transformation of time (ω = 0.88)**		
8. Time seemed to alter (either slowed down or speeded up)	0.74	0.83
17. The way time passed seemed to be different from normal	0.82	0.91
26. It felt like time went by quickly	0.64	0.6
35. I lost my normal awareness of time	0.74	0.82
**Autotelic experience (ω = 0.86)**		
9. I really enjoyed the experience of what I was doing	0.81	0.82
18. I loved the feeling of what I was doing, and want to capture this feeling again	0.82	0.82
27. The experience left me feeling great	0.93	0.92
36. I found the experience extremely rewarding	0.66	0.65
**Global flow factor (ω = 0.85)**		
Challenge skill balance	0.86	
Action and awareness	0.54	
Clear goals	0.91	
Unambiguous feedback	0.85	
Task concentration	0.65	
Sense of control	0.92	
Loss of self-consciousness	0.40	
Transformation of time	0.10	
Autotelic experience	0.62	
		

### Reliability Analysis of the Flow State Scale-2

All factors tested in the analysis showed good reliability, as indicated by ω ≥ 0.7 (Table 4).

Additionally, intercorrelations of items of the FSS-2 questionnaire are presented in the Supplementary Material.

The results of the analyses in this study provided strong support for the validity and reliability of the Polish version of Dispositional Flow Scale-2 (PDFS-2) and the Polish version of Flow State Scale-2 (PFSS-2) in assessing the experience of flow in the activity of playing games as entertainment among adult Poles.

As hypothesized, the psychometric properties obtained with the validated Polish versions of the DFS-2 and FFS-2 overlap with the responses of adult Polish speakers.

The maximum likelihood estimator (MLR) was selected for CFA analysis. Model fit was assessed using indices: χ2, comparative fit index (CFI), Tucker-Lewis index (TLI), and standardized root mean squares of residuals (SRMR) and root mean squares of approximation errors (RMSEA).

For both the DFS-2 and FSS-2 questionnaires, formative first-level models with nine factors and second-level models with nine factors loaded on a higher-order flow factor were compared using the Satorra-Bentler Scaled difference χ2 test.

The ω coefficient was used to estimate the reliability of the FFS-2 and DFS-2 models tested using the CFA method.

#### DFS-2

##### Confirmatory Factor Analysis of the Dispositional Flow Scale-2

Both DFS-2 structural models showed satisfactory model fit, but the metrics were better for the DFS-2 first order model. Furthermore, the scaled Satorra-Bentler χ2 difference showed a significant difference between the models, χ2 difference = 162.91, *p* < 0.001, favoring the formative first order model with nine factors.

##### Reliability Analysis of the Dispositional Flow Scale-2 Questionnaire

All factors examined in the analysis showed good reliability, as indicated by ω ≥ 0.7.

#### FSS-2

##### Confirmatory Factor Analysis of the Dispositional Flow Status Scale-2

Most of the fit indices for the hierarchical FSS-2 second order model presented satisfactory values, except for SRMR. The model parameters were examined to find the cause of the misfit. For the hierarchical model, the “Time Transformation” factor was not significantly loaded by the global flow factor (*r* = 0.10, *p* = 0.150). The first order FSS-2 model showed better values for all fit indices. Additionally, the scaled Satorra-Bentler χ2 difference showed a significant difference between the models, χ2 difference = 92.00, *p* < 0.001, favoring the formative first order model with nine factors.

##### Reliability Analysis of the Flow State Scale-2

All factors examined in the analysis showed good reliability, as indicated by ω ≥ 0.7.

Intercorrelations of DFS-2 and FSS-2 questionnaire items are presented in the Supplementary Material.

## Discussion

The main aim of the present study was to assess the psychometric properties of the Polish version of DFS-2 and FSS-2 for use with Polish adults. For this purpose, following the principles of test adaptation proposed by Gawlik and Kurpas (2014), a diversified group of participants from all over the country, from large and small towns and villages of various ages, was invited to the study. Due to the limitations of COVID-19, the recruitment and the study itself took place online, hence the participation required Internet access, which is a limitation.

Thanks to the research described above, it was possible to create a solid adaptation of PDFS-2 and PFFS-2 for adults speaking Polish.

The study of flow allows us to understand experiences during which individuals are fully engaged in the present moment of a task. Flow is a state that occurs during full engagement, in which the individual is operating at a level consistent with the demands of the task and is fully focused on the present moment, which positive psychology defines as one of the key determinants of a good, happy life. Flow, as a phenomenon potentially present in every area of life, has been studied in sports, music, chess, dance and games, including video games, among others. This tool validation was prepared for the research project “Player playability. Correlation between the dynamics of changes in cognitive functioning resulting from complex skill learning through play and player flow, motivation and perceived playability.” The purpose of this project is, among other things, to verify how a person’s flow influences their learning process of a complex game. The flow measurement tools will allow for a better understanding of the influence of video games on cognitive functions and the mediators that accompany this process. The starting point for this project is the concept of flow, which is the assumption that humans function optimally when the challenges they face are balanced (Csíkszentmihályi, 1990; Moneta and Csíkszentmihályi, 1996). This allows us to assume that the development of cognitive functions will proceed more efficiently in a higher flow state.

### Limitations and Methodological Considerations

The results of the study may be affected by the fact that the participants of the study carried out their activity with the awareness of the participation in the study–this was the first activity carried out in a group of people who did not know each other.

Another limitation may be the fact that the research was performed online due to the limitations of COVID-19.

## Conclusion

The current research has provided indications that both the Polish versions of DFS-2 and FSS-2 are valid and reliable questionnaires. During the study, several aspects emerged that should be examined in future research in order to better understand both the nature of the flow phenomenon and the questionnaire tools themselves.

Possible directions of exploration:

1. Criteria-related validity (Kline, 2005) using self-report tools of other psychological constructs (motivation, immersion, commitment, self-esteem, locus of control) or variables (participation and skills levels). Some of them (motivation, locus of control, skills levels) are part of the “Playability of the Player” study.
2. Verification of the stability of the questionnaires. It seems interesting to verify to what extent the measurement for a given participant is stable. While the nature of the FSS-2 tool implies that it is related to the “here and now,” it would be interesting to observe whether there are trends within a person’s measure. A study carried out by the team of authors of this article, “Playability of the player,” which assumes repeated measurement of flow as a state, spread over time, and related to learning a specific complex activity, may be helpful here. In the context of the DFS-2, it is worth looking at its stability, which Kawabata et al. (2008) undertook by measuring it twice over a 4-week interval. In the cited study, factors such as task focus, loss of self-awareness, time transformation, and autotelic experience showed slightly less stability than others. However, because the stability of responses to DFS-2 was not tested, it is not possible to determine whether these characteristics are sample specific.
3. Verification of the correlation between DFS-2 and FSS-2. Another interesting direction seems to be the implementation of group studies using both questionnaires (DFS-2 and FSS-2). Also such an activity has been planned in the framework of the project “Playability of the player,” where the participants first complete the DFS-2 questionnaire and then take part in a learning process with repeated measurement using the FSS-2 questionnaire.

The scales assessed in this study are useful tools for a variety of research purposes. They are also relevant to those studying flow in various aspects of life, including video games, which provide a safe yet compelling and variedly challenging environment for exploration (Wang et al., 2009; Procci et al., 2012; Hamari and Koivisto, 2014; Gao and Lu, 2021; Gutierrez, 2021; Cai et al., 2022; Jogo et al., 2022, etc.).

The results of the study allow for the use of the Polish version of the tool, which will make it possible to study flow using the DFS-2 and FSS-2 in both game studies and other activities where the potential for flow state is identified.

## Data Availability Statement

The original contributions presented in the study are included in the article/Supplementary Material, further inquiries can be directed to the corresponding authors.

## Ethics Statement

The studies involving human participants were reviewed and approved by Commission on Research Ethics of the Faculty of Psychology, SWPS University in Warsaw. The patients/participants provided their written informed consent to participate in this study.

## Author Contributions

JJ and NK-G conceptualized the project. JJ conducted data analyses, verified by AB and NK-G. All authors take full responsibility for the integrity and accuracy of the data analysis and approved the final version for submission.

## Conflict of Interest

The authors declare that the research was conducted in the absence of any commercial or financial relationships that could be construed as a potential conflict of interest.

## Publisher’s Note

All claims expressed in this article are solely those of the authors and do not necessarily represent those of their affiliated organizations, or those of the publisher, the editors and the reviewers. Any product that may be evaluated in this article, or claim that may be made by its manufacturer, is not guaranteed or endorsed by the publisher.
