# Supplementary material for: Validation of Polish Version of Dispositional Flow Scale-2 and Flow State Scale-2 Questionnaires
Source: Front Psychol. 2022 Apr 25;13:818036. doi: 10.3389/fpsyg.2022.818036 (PMC9082954; doi:10.3389/fpsyg.2022.818036)
Supplement: Supplementary file 1 [file Data_Sheet_1.PDF]

Supplementary Table 1

Intercorrelations between DFS-2 items

dfs1	dfs2	dfs3	dfs4	dfs5	dfs6	dfs7	dfs8	dfs9	dfs10	dfs11	dfs12	dfs13	dfs14	dfs15	dfs16	dfs17	dfs18	dfs19	dfs20	dfs21	dfs22	dfs23	dfs24	dfs25	dfs26	dfs27	dfs28	dfs29	dfs30	dfs31	dfs32	dfs33	dfs34	dfs35	dfs36
1	0.28	0.42	0.39	0.34	0.45	0.25	0.17	0.39	0.48	0.23	0.4	0.41	0.25	0.47	0.24	0.17	0.38	0.49	0.14	0.42	0.42	0.25	0.4	0.16	0.13	0.37	0.46	0.25	0.37	0.36	0.34	0.41	0.22	0.11	0.41
0.28	1	0.28	0.26	0.16	0.31	0.22	0.07	0.13	0.22	0.26	0.24	0.27	0.29	0.31	0.23	0.1	0.18	0.29	0.38	0.17	0.27	0.11	0.28	0.13	0.11	0.14	0.23	0.45	0.18	0.24	0.13	0.29	0.21	0.09	0.17
0.42	0.28	1	0.53	0.4	0.5	0.26	0.05	0.34	0.44	0.24	0.67	0.44	0.32	0.49	0.23	0.09	0.37	0.46	0.18	0.57	0.46	0.39	0.52	0.24	0.2	0.36	0.4	0.29	0.56	0.37	0.43	0.46	0.23	0.06	0.38
0.39	0.26	0.53	1	0.38	0.48	0.29	0.1	0.31	0.39	0.23	0.49	0.59	0.28	0.44	0.3	0.09	0.23	0.44	0.24	0.45	0.6	0.36	0.5	0.27	0.15	0.27	0.43	0.28	0.42	0.45	0.39	0.46	0.28	0.07	0.33
0.34	0.16	0.4	0.38	1	0.48	0.22	0.16	0.31	0.32	0.19	0.38	0.31	0.24	0.36	0.17	0.21	0.31	0.3	0.05	0.36	0.34	0.63	0.48	0.25	0.13	0.3	0.32	0.16	0.42	0.29	0.67	0.44	0.24	0.14	0.39
0.45	0.31	0.5	0.48	0.48	1	0.29	0.08	0.39	0.41	0.2	0.51	0.38	0.33	0.58	0.24	0.11	0.34	0.45	0.17	0.43	0.4	0.47	0.6	0.22	0.17	0.36	0.4	0.28	0.46	0.33	0.49	0.54	0.23	0.1	0.39
0.25	0.22	0.26	0.29	0.22	0.29	1	0.13	0.2	0.21	0.18	0.29	0.3	0.28	0.29	0.82	0.18	0.21	0.3	0.31	0.26	0.31	0.21	0.35	0.62	0.16	0.24	0.26	0.36	0.25	0.26	0.28	0.26	0.71	0.16	0.27
0.17	0.07	0.05	0.1	0.16	0.08	0.13	1	0.17	0.18	0.19	0.04	0.1	0.11	0.06	0.15	0.64	0.2	0.14	0.06	0.08	0.07	0.12	0.07	0.15	0.48	0.22	0.13	0.07	0.1	0.18	0.14	0.08	0.15	0.51	0.21
0.39	0.13	0.34	0.31	0.31	0.39	0.2	0.17	1	0.42	0.26	0.42	0.36	0.28	0.38	0.21	0.24	0.61	0.44	0.15	0.44	0.32	0.31	0.36	0.18	0.2	0.61	0.35	0.24	0.39	0.33	0.34	0.34	0.2	0.21	0.56
0.48	0.22	0.44	0.39	0.32	0.41	0.21	0.18	0.42	1	0.31	0.51	0.52	0.27	0.45	0.23	0.15	0.37	0.63	0.17	0.43	0.42	0.31	0.43	0.18	0.2	0.38	0.57	0.26	0.42	0.44	0.39	0.46	0.25	0.09	0.37
0.23	0.26	0.24	0.23	0.19	0.2	0.18	0.19	0.26	0.31	1	0.24	0.21	0.29	0.29	0.2	0.23	0.27	0.32	0.41	0.25	0.27	0.15	0.24	0.19	0.31	0.31	0.26	0.43	0.25	0.32	0.21	0.27	0.22	0.28	0.26
0.4	0.24	0.67	0.49	0.38	0.51	0.29	0.04	0.42	0.51	0.24	1	0.51	0.35	0.5	0.31	0.11	0.44	0.54	0.17	0.7	0.47	0.4	0.49	0.26	0.21	0.46	0.48	0.29	0.64	0.41	0.45	0.49	0.31	0.13	0.47
0.41	0.27	0.44	0.59	0.31	0.38	0.3	0.1	0.36	0.52	0.21	0.51	1	0.35	0.54	0.37	0.18	0.33	0.57	0.24	0.47	0.65	0.34	0.5	0.27	0.15	0.34	0.47	0.32	0.47	0.57	0.38	0.51	0.35	0.07	0.32
0.25	0.29	0.32	0.28	0.24	0.33	0.28	0.11	0.28	0.27	0.29	0.35	0.35	1	0.44	0.29	0.19	0.36	0.38	0.32	0.3	0.31	0.3	0.33	0.3	0.16	0.33	0.31	0.38	0.32	0.28	0.33	0.35	0.32	0.19	0.31
0.47	0.31	0.49	0.44	0.36	0.58	0.29	0.06	0.38	0.45	0.29	0.5	0.54	0.44	1	0.32	0.11	0.4	0.58	0.24	0.51	0.51	0.41	0.64	0.24	0.15	0.43	0.46	0.38	0.46	0.42	0.46	0.64	0.32	0.1	0.41
0.24	0.23	0.23	0.3	0.17	0.24	0.82	0.15	0.21	0.23	0.2	0.31	0.37	0.29	0.32	1	0.24	0.23	0.33	0.33	0.3	0.34	0.2	0.32	0.6	0.16	0.24	0.26	0.36	0.27	0.31	0.29	0.32	0.75	0.16	0.25
0.17	0.1	0.09	0.09	0.21	0.11	0.18	0.64	0.24	0.15	0.23	0.11	0.18	0.19	0.11	0.24	1	0.29	0.17	0.12	0.1	0.13	0.21	0.12	0.2	0.48	0.31	0.15	0.15	0.12	0.25	0.19	0.16	0.24	0.61	0.31
0.38	0.18	0.37	0.23	0.31	0.34	0.21	0.2	0.61	0.37	0.27	0.44	0.33	0.36	0.4	0.23	0.29	1	0.53	0.19	0.42	0.3	0.32	0.37	0.22	0.22	0.68	0.38	0.29	0.4	0.31	0.37	0.38	0.22	0.3	0.68
0.49	0.29	0.46	0.44	0.3	0.45	0.3	0.14	0.44	0.63	0.32	0.54	0.57	0.38	0.58	0.33	0.17	0.53	1	0.25	0.53	0.52	0.35	0.53	0.26	0.21	0.5	0.62	0.35	0.5	0.5	0.39	0.54	0.3	0.16	0.49
0.14	0.38	0.18	0.24	0.05	0.17	0.31	0.06	0.15	0.17	0.41	0.17	0.24	0.32	0.24	0.33	0.12	0.19	0.25	1	0.19	0.27	0.09	0.26	0.25	0.2	0.19	0.28	0.64	0.17	0.27	0.13	0.26	0.31	0.19	0.16
0.42	0.17	0.57	0.45	0.36	0.43	0.26	0.08	0.44	0.43	0.25	0.7	0.47	0.3	0.51	0.3	0.1	0.42	0.53	0.19	1	0.56	0.43	0.51	0.22	0.2	0.47	0.46	0.3	0.69	0.46	0.47	0.52	0.3	0.17	0.47
0.42	0.27	0.46	0.6	0.34	0.4	0.31	0.07	0.32	0.42	0.27	0.47	0.65	0.31	0.51	0.34	0.13	0.3	0.52	0.27	0.56	1	0.39	0.55	0.25	0.17	0.33	0.47	0.33	0.52	0.61	0.45	0.53	0.33	0.08	0.32
0.25	0.11	0.39	0.36	0.63	0.47	0.21	0.12	0.31	0.31	0.15	0.4	0.34	0.3	0.41	0.2	0.21	0.32	0.35	0.09	0.43	0.39	1	0.55	0.28	0.15	0.4	0.35	0.19	0.48	0.33	0.73	0.49	0.27	0.17	0.41
0.4	0.28	0.52	0.5	0.48	0.6	0.35	0.07	0.36	0.43	0.24	0.49	0.5	0.33	0.64	0.32	0.12	0.37	0.53	0.26	0.51	0.55	0.55	1	0.34	0.12	0.4	0.54	0.41	0.55	0.43	0.57	0.66	0.38	0.1	0.44
0.16	0.13	0.24	0.27	0.25	0.22	0.62	0.15	0.18	0.18	0.19	0.26	0.27	0.3	0.24	0.6	0.2	0.22	0.26	0.25	0.22	0.25	0.28	0.34	1	0.14	0.26	0.25	0.34	0.26	0.19	0.28	0.26	0.66	0.19	0.26
0.13	0.11	0.2	0.15	0.13	0.17	0.16	0.48	0.2	0.2	0.31	0.21	0.15	0.16	0.15	0.16	0.48	0.22	0.21	0.2	0.2	0.17	0.15	0.12	0.14	1	0.29	0.19	0.18	0.2	0.24	0.15	0.19	0.12	0.52	0.2
0.37	0.14	0.36	0.27	0.3	0.36	0.24	0.22	0.61	0.38	0.31	0.46	0.34	0.33	0.43	0.24	0.31	0.68	0.5	0.19	0.47	0.33	0.4	0.4	0.26	0.29	1	0.41	0.34	0.46	0.35	0.39	0.38	0.27	0.31	0.72
0.46	0.23	0.4	0.43	0.32	0.4	0.26	0.13	0.35	0.57	0.26	0.48	0.47	0.31	0.46	0.26	0.15	0.38	0.62	0.28	0.46	0.47	0.35	0.54	0.25	0.19	0.41	1	0.41	0.5	0.42	0.41	0.5	0.29	0.13	0.38
0.25	0.45	0.29	0.28	0.16	0.28	0.36	0.07	0.24	0.26	0.43	0.29	0.32	0.38	0.38	0.36	0.15	0.29	0.35	0.64	0.3	0.33	0.19	0.41	0.34	0.18	0.34	0.41	1	0.32	0.28	0.23	0.35	0.39	0.18	0.28
0.37	0.18	0.56	0.42	0.42	0.46	0.25	0.1	0.39	0.42	0.25	0.64	0.47	0.32	0.46	0.27	0.12	0.4	0.5	0.17	0.69	0.52	0.48	0.55	0.26	0.2	0.46	0.5	0.32	1	0.49	0.5	0.52	0.3	0.13	0.45
0.36	0.24	0.37	0.45	0.29	0.33	0.26	0.18	0.33	0.44	0.32	0.41	0.57	0.28	0.42	0.31	0.25	0.31	0.5	0.27	0.46	0.61	0.33	0.43	0.19	0.24	0.35	0.42	0.28	0.49	1	0.42	0.47	0.29	0.21	0.34
0.34	0.13	0.43	0.39	0.67	0.49	0.28	0.14	0.34	0.39	0.21	0.45	0.38	0.33	0.46	0.29	0.19	0.37	0.39	0.13	0.47	0.45	0.73	0.57	0.28	0.15	0.39	0.41	0.23	0.5	0.42	1	0.6	0.31	0.19	0.45
0.41	0.29	0.46	0.46	0.44	0.54	0.26	0.08	0.34	0.46	0.27	0.49	0.51	0.35	0.64	0.32	0.16	0.38	0.54	0.26	0.52	0.53	0.49	0.66	0.26	0.19	0.38	0.5	0.35	0.52	0.47	0.6	1	0.38	0.14	0.42
0.22	0.21	0.23	0.28	0.24	0.23	0.71	0.15	0.2	0.25	0.22	0.31	0.35	0.32	0.32	0.75	0.24	0.22	0.3	0.31	0.3	0.33	0.27	0.38	0.66	0.12	0.27	0.29	0.39	0.3	0.29	0.31	0.38	1	0.16	0.26
0.11	0.09	0.06	0.07	0.14	0.1	0.16	0.51	0.21	0.09	0.28	0.13	0.07	0.19	0.1	0.16	0.61	0.3	0.16	0.19	0.17	0.08	0.17	0.1	0.19	0.52	0.31	0.13	0.18	0.13	0.21	0.19	0.14	0.16	1	0.29
0.41	0.17	0.38	0.33	0.39	0.39	0.27	0.21	0.56	0.37	0.26	0.47	0.32	0.31	0.41	0.25	0.31	0.68	0.49	0.16	0.47	0.32	0.41	0.44	0.26	0.2	0.72	0.38	0.28	0.45	0.34	0.45	0.42	0.26	0.29	1
