# Supplementary material for: Validation of Polish Version of Dispositional Flow Scale-2 and Flow State Scale-2 Questionnaires
Source: Front Psychol. 2022 Apr 25;13:818036. doi: 10.3389/fpsyg.2022.818036 (PMC9082954; doi:10.3389/fpsyg.2022.818036)
Supplement: Supplementary file 2 [file Data_Sheet_2.PDF]

Supplementary Table 2

Intercorrelations between FSS-2 items

fss1	fss2	fss3	fss4	fss5	fss6	fss7	fss8	fss9	fss10	fss11	fss12	fss13	fss14	fss15	fss16	fss17	fss18	fss19	fss20	fss21	fss22	fss23	fss24	fss25	fss26	fss27	fss28	fss29	fss30	fss31	fss32	fss33	fss34	fss35	fss36
1	0.3	0.41	0.34	0.36	0.42	0.16	0	0.45	0.57	0.25	0.39	0.39	0.16	0.45	0.12	-0.02	0.31	0.53	0.19	0.41	0.39	0.33	0.44	0.13	0.2	0.4	0.56	0.2	0.38	0.36	0.32	0.39	0.17	0.07	0.26
0.3	1	0.38	0.29	0.19	0.36	0.18	0.13	0.22	0.41	0.33	0.4	0.39	0.4	0.42	0.17	0.13	0.15	0.38	0.44	0.31	0.37	0.16	0.41	0.11	0.17	0.21	0.34	0.47	0.29	0.27	0.19	0.49	0.17	0.17	0.09
0.41	0.38	1	0.56	0.4	0.55	0.23	-0.02	0.38	0.46	0.23	0.7	0.5	0.25	0.51	0.2	0.03	0.3	0.51	0.21	0.6	0.52	0.39	0.56	0.17	0.24	0.39	0.44	0.34	0.53	0.39	0.45	0.59	0.23	0.08	0.17
0.34	0.29	0.56	1	0.28	0.49	0.22	-0.08	0.34	0.41	0.18	0.56	0.62	0.26	0.52	0.21	-0.06	0.22	0.47	0.16	0.47	0.65	0.26	0.55	0.18	0.14	0.33	0.42	0.26	0.47	0.53	0.31	0.51	0.2	0.07	0.19
0.36	0.19	0.4	0.28	1	0.51	0.26	0.21	0.49	0.32	0.16	0.37	0.33	0.09	0.37	0.14	0.16	0.41	0.37	0.09	0.44	0.35	0.72	0.38	0.17	0.26	0.46	0.36	0.14	0.4	0.36	0.73	0.41	0.21	0.19	0.22
0.42	0.36	0.55	0.49	0.51	1	0.24	0.06	0.44	0.46	0.19	0.58	0.49	0.25	0.66	0.2	0.03	0.34	0.5	0.21	0.51	0.51	0.44	0.67	0.16	0.22	0.37	0.42	0.27	0.5	0.44	0.5	0.65	0.2	0.12	0.15
0.16	0.18	0.23	0.22	0.26	0.24	1	0	0.15	0.21	0.13	0.32	0.33	0.14	0.27	0.78	0.06	0.07	0.25	0.22	0.25	0.26	0.32	0.34	0.67	0.09	0.18	0.19	0.2	0.28	0.23	0.29	0.34	0.78	0.09	0.12
0	0.13	-0.02	-0.08	0.21	0.06	0	1	0.18	-0.01	0.16	0	-0.06	0.1	0	-0.02	0.78	0.1	-0.05	0.08	0.07	-0.02	0.12	0	-0.03	0.44	0.09	0.02	0.05	0.03	0.06	0.13	0.02	-0.03	0.66	0.1
0.45	0.22	0.38	0.34	0.49	0.44	0.15	0.18	1	0.48	0.24	0.38	0.34	0.21	0.42	0.13	0.16	0.67	0.54	0.12	0.47	0.36	0.46	0.48	0.12	0.4	0.74	0.57	0.21	0.4	0.44	0.46	0.43	0.14	0.29	0.53
0.57	0.41	0.46	0.41	0.32	0.46	0.21	-0.01	0.48	1	0.37	0.56	0.53	0.25	0.59	0.18	-0.03	0.33	0.79	0.33	0.54	0.55	0.31	0.59	0.17	0.19	0.46	0.65	0.33	0.49	0.44	0.37	0.59	0.22	0.05	0.35
0.25	0.33	0.23	0.18	0.16	0.19	0.13	0.16	0.24	0.37	1	0.3	0.17	0.21	0.3	0.1	0.17	0.15	0.32	0.45	0.3	0.23	0.15	0.29	0.11	0.23	0.21	0.25	0.43	0.27	0.23	0.2	0.25	0.15	0.22	0.12
0.39	0.4	0.7	0.56	0.37	0.58	0.32	0	0.38	0.56	0.3	1	0.61	0.31	0.59	0.26	0	0.29	0.55	0.29	0.66	0.61	0.38	0.66	0.25	0.22	0.36	0.43	0.31	0.6	0.47	0.45	0.59	0.28	0.07	0.2
0.39	0.39	0.5	0.62	0.33	0.49	0.33	-0.06	0.34	0.53	0.17	0.61	1	0.31	0.56	0.24	0.01	0.26	0.57	0.26	0.5	0.72	0.32	0.57	0.23	0.19	0.33	0.47	0.32	0.48	0.6	0.34	0.59	0.29	0.05	0.17
0.16	0.4	0.25	0.26	0.09	0.25	0.14	0.1	0.21	0.25	0.21	0.31	0.31	1	0.37	0.13	0.15	0.12	0.3	0.35	0.21	0.3	0.11	0.32	0.14	0.26	0.15	0.25	0.41	0.19	0.18	0.19	0.3	0.15	0.19	0.09
0.45	0.42	0.51	0.52	0.37	0.66	0.27	0	0.42	0.59	0.3	0.59	0.56	0.37	1	0.24	-0.02	0.29	0.65	0.28	0.52	0.59	0.38	0.75	0.21	0.18	0.36	0.45	0.37	0.51	0.49	0.42	0.7	0.26	0.06	0.18
0.12	0.17	0.2	0.21	0.14	0.2	0.78	-0.02	0.13	0.18	0.1	0.26	0.24	0.13	0.24	1	0.04	0.06	0.21	0.25	0.22	0.23	0.24	0.33	0.71	0.11	0.16	0.21	0.23	0.24	0.21	0.25	0.31	0.79	0.08	0.1
-0.02	0.13	0.03	-0.06	0.16	0.03	0.06	0.78	0.16	-0.03	0.17	0	0.01	0.15	-0.02	0.04	1	0.14	-0.01	0.13	0.09	-0.03	0.13	-0.01	0.06	0.5	0.11	0.07	0.18	0.04	0.06	0.12	0.08	0.09	0.74	0.09
0.31	0.15	0.3	0.22	0.41	0.34	0.07	0.1	0.67	0.33	0.15	0.29	0.26	0.12	0.29	0.06	0.14	1	0.41	0.09	0.42	0.28	0.33	0.37	0.07	0.34	0.77	0.47	0.15	0.35	0.29	0.35	0.33	0.11	0.19	0.52
0.53	0.38	0.51	0.47	0.37	0.5	0.25	-0.05	0.54	0.79	0.32	0.55	0.57	0.3	0.65	0.21	-0.01	0.41	1	0.31	0.58	0.6	0.34	0.64	0.23	0.21	0.51	0.68	0.37	0.54	0.48	0.42	0.63	0.27	0.08	0.32
0.19	0.44	0.21	0.16	0.09	0.21	0.22	0.08	0.12	0.33	0.45	0.29	0.26	0.35	0.28	0.25	0.13	0.09	0.31	1	0.25	0.27	0.17	0.31	0.2	0.12	0.15	0.22	0.69	0.21	0.17	0.17	0.3	0.29	0.16	0.04
0.41	0.31	0.6	0.47	0.44	0.51	0.25	0.07	0.47	0.54	0.3	0.66	0.5	0.21	0.52	0.22	0.09	0.42	0.58	0.25	1	0.62	0.43	0.55	0.23	0.23	0.49	0.54	0.28	0.73	0.54	0.48	0.56	0.24	0.13	0.32
0.39	0.37	0.52	0.65	0.35	0.51	0.26	-0.02	0.36	0.55	0.23	0.61	0.72	0.3	0.59	0.23	-0.03	0.28	0.6	0.27	0.62	1	0.34	0.6	0.19	0.16	0.35	0.49	0.31	0.56	0.65	0.4	0.6	0.22	0.07	0.22
0.33	0.16	0.39	0.26	0.72	0.44	0.32	0.12	0.46	0.31	0.15	0.38	0.32	0.11	0.38	0.24	0.13	0.33	0.34	0.17	0.43	0.34	1	0.43	0.24	0.29	0.45	0.37	0.21	0.4	0.35	0.79	0.43	0.27	0.16	0.2
0.44	0.41	0.56	0.55	0.38	0.67	0.34	0	0.48	0.59	0.29	0.66	0.57	0.32	0.75	0.33	-0.01	0.37	0.64	0.31	0.55	0.6	0.43	1	0.29	0.22	0.46	0.55	0.4	0.56	0.46	0.51	0.76	0.32	0.08	0.29
0.13	0.11	0.17	0.18	0.17	0.16	0.67	-0.03	0.12	0.17	0.11	0.25	0.23	0.14	0.21	0.71	0.06	0.07	0.23	0.2	0.23	0.19	0.24	0.29	1	0.12	0.15	0.19	0.26	0.24	0.21	0.24	0.31	0.77	0.11	0.12
0.2	0.17	0.24	0.14	0.26	0.22	0.09	0.44	0.4	0.19	0.23	0.22	0.19	0.26	0.18	0.11	0.5	0.34	0.21	0.12	0.23	0.16	0.29	0.22	0.12	1	0.36	0.26	0.22	0.27	0.23	0.29	0.23	0.16	0.58	0.24
0.4	0.21	0.39	0.33	0.46	0.37	0.18	0.09	0.74	0.46	0.21	0.36	0.33	0.15	0.36	0.16	0.11	0.77	0.51	0.15	0.49	0.35	0.45	0.46	0.15	0.36	1	0.6	0.23	0.43	0.39	0.44	0.42	0.2	0.2	0.62
0.56	0.34	0.44	0.42	0.36	0.42	0.19	0.02	0.57	0.65	0.25	0.43	0.47	0.25	0.45	0.21	0.07	0.47	0.68	0.22	0.54	0.49	0.37	0.55	0.19	0.26	0.6	1	0.32	0.46	0.45	0.41	0.49	0.25	0.17	0.38
0.2	0.47	0.34	0.26	0.14	0.27	0.2	0.05	0.21	0.33	0.43	0.31	0.32	0.41	0.37	0.23	0.18	0.15	0.37	0.69	0.28	0.31	0.21	0.4	0.26	0.22	0.23	0.32	1	0.26	0.2	0.25	0.38	0.31	0.2	0.05
0.38	0.29	0.53	0.47	0.4	0.5	0.28	0.03	0.4	0.49	0.27	0.6	0.48	0.19	0.51	0.24	0.04	0.35	0.54	0.21	0.73	0.56	0.4	0.56	0.24	0.27	0.43	0.46	0.26	1	0.53	0.48	0.55	0.31	0.06	0.3
0.36	0.27	0.39	0.53	0.36	0.44	0.23	0.06	0.44	0.44	0.23	0.47	0.6	0.18	0.49	0.21	0.06	0.29	0.48	0.17	0.54	0.65	0.35	0.46	0.21	0.23	0.39	0.45	0.2	0.53	1	0.4	0.5	0.23	0.12	0.27
0.32	0.19	0.45	0.31	0.73	0.5	0.29	0.13	0.46	0.37	0.2	0.45	0.34	0.19	0.42	0.25	0.12	0.35	0.42	0.17	0.48	0.4	0.79	0.51	0.24	0.29	0.44	0.41	0.25	0.48	0.4	1	0.47	0.29	0.14	0.21
0.39	0.49	0.59	0.51	0.41	0.65	0.34	0.02	0.43	0.59	0.25	0.59	0.59	0.3	0.7	0.31	0.08	0.33	0.63	0.3	0.56	0.6	0.43	0.76	0.31	0.23	0.42	0.49	0.38	0.55	0.5	0.47	1	0.37	0.13	0.26
0.17	0.17	0.23	0.2	0.21	0.2	0.78	-0.03	0.14	0.22	0.15	0.28	0.29	0.15	0.26	0.79	0.09	0.11	0.27	0.29	0.24	0.22	0.27	0.32	0.77	0.16	0.2	0.25	0.31	0.31	0.23	0.29	0.37	1	0.1	0.16
0.07	0.17	0.08	0.07	0.19	0.12	0.09	0.66	0.29	0.05	0.22	0.07	0.05	0.19	0.06	0.08	0.74	0.19	0.08	0.16	0.13	0.07	0.16	0.08	0.11	0.58	0.2	0.17	0.2	0.06	0.12	0.14	0.13	0.1	1	0.19
0.26	0.09	0.17	0.19	0.22	0.15	0.12	0.1	0.53	0.35	0.12	0.2	0.17	0.09	0.18	0.1	0.09	0.52	0.32	0.04	0.32	0.22	0.2	0.29	0.12	0.24	0.62	0.38	0.05	0.3	0.27	0.21	0.26	0.16	0.19	1
